# P-205. Investigating PCR (Polymerase Chain Reaction) Cycle Threshold’s Utility in Diagnosing and Treating Clostridioides difficile Infections

**DOI:** 10.1093/ofid/ofae631.409

**Published:** 2025-01-29

**Authors:** Edward Carey, Bennadette Maramara, Matthew Jacob, Arthur H Totten, Roderick Go

**Affiliations:** Stony Brook University Renaissance School of Medicine, Mineola, New York; Stony Brook University Hospital, stony brook, New York; Stony Brook University Hospital, stony brook, New York; Stony Brook Medicine, Stony Brook, New York; Stony Brook University Hospital, stony brook, New York

## Abstract

**Background:**

*Clostridioides difficile* (C diff) is a pathobiont bacteria in the human gastrointestinal tract that can cause morbidity and mortality and has public health implications.

Guidelines suggest two-step testing to diagnose C diff infection (CDI). However, since polymerase chain reaction (PCR) tests can be positive in persons with colonization and no active disease, management of discordant results is challenging. Usage of facility-onset CDI as a quality metric further raises the need to accurately differentiate infection from colonization.

We hypothesize PCR cycle threshold (Ct) values may be useful for quantifying C diff disease burden and differentiation of infection from colonization. However, Cts are currently unvalidated and are not currently approved for use for clinical decision making.

Sample and results data for C diff infection and C diff PCR CT correlation

**Figure:**
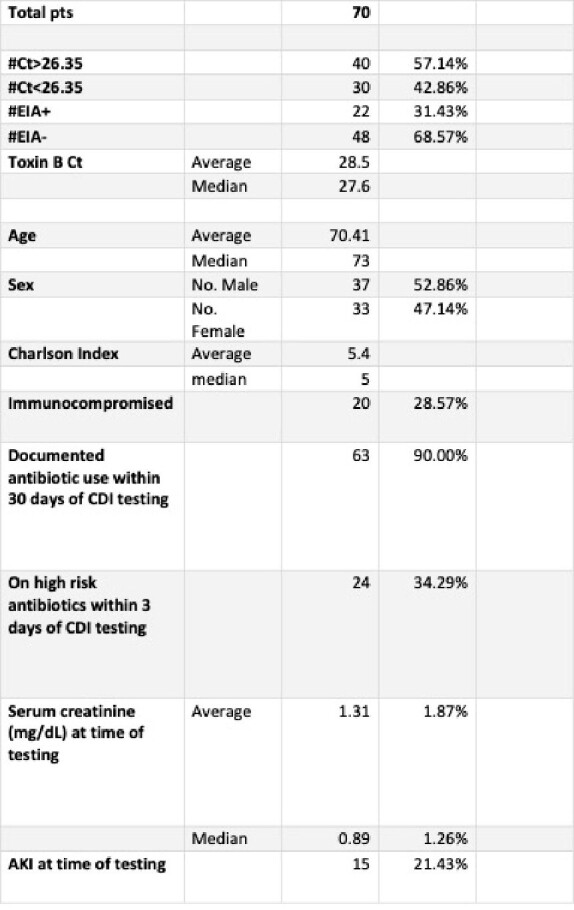

This table provides an overview of our data investigating the usage of C diff PCR CT to better diagnose CDI

**Methods:**

We performed a retrospective, single center chart review of patients who tested positive for C diff, and analyzed clinical and laboratory data in 2022. We excluded minors, outpatients, and repeat C diff episodes, and performed multi variable analyses.

**Results:**

70 patients met inclusion criteria. This cohort consisted of 37 (52.86%) male and 33 (47%) female patients. The mean and median ages were 70 and 73 years old respectively. Approximately 30 percent of our cohort was immunocompromised, and 90 percent of our patients were on antibiotics within 30 days of CDI testing.

Using a Ct of 26.35, a cutoff used in previous studies to indicate infection, 30 patients (42.9%) had a positive Ct, and of these, 16 (53%), had a positive confirmatory enzyme immunoassay (EIA) . Ct of less than cutoff had a positive predictive value of 53%, and negative predictive value of 85%, for positive EIA.

All patients analyzed underwent C diff treatment, with a mean length of treatment of 10 days. Mean time to recovery, classified as less than three documented bowel movements, or hospital discharge, was 4.6 days. Nine patients (13%) in our cohort died within 30 days of C diff testing.

**Conclusion:**

This data show that C diff PCR Ct value threshold of < 26.35 combined with EIA positivity may be a helpful parameter for evaluating possible C diff. The demonstrated high negative predictive value may show a utility for high Ct values in ruling out active disease.

**Disclosures:**

**Roderick Go, DO**, Aptose Biosciences: Stocks/Bonds (Private Company)|Bristol Meyers Squibb: Stocks/Bonds (Private Company)|Cytodyn Inc.: Stocks/Bonds (Private Company)|Scynexis: Grant/Research Support|Shionogi, Inc: Grant/Research Support

